# Hypocholesterolemic and Prebiotic Effects of a Whole-Grain Oat-Based Granola Breakfast Cereal in a Cardio-Metabolic “At Risk” Population

**DOI:** 10.3389/fmicb.2016.01675

**Published:** 2016-11-07

**Authors:** Michael L. Connolly, Xenofon Tzounis, Kieran M. Tuohy, Julie A. Lovegrove

**Affiliations:** ^1^Hugh Sinclair Unit of Human Nutrition, University of ReadingReading, UK; ^2^Institute for Cardiovascular and Metabolic Research, University of ReadingReading, UK; ^3^Department of Food Quality and Nutrition, Research and Innovation Centre - Fondazione Edmund MachTrento, Italy

**Keywords:** whole-grain oat granola, prebiotic, cholesterol, cardiovascular risk, *Bifidobacterium*

## Abstract

Meta-analyses of randomized controlled trials (RTC) have confirmed the hypocholesterolaemic effect of oats and oat based fibers. However, the mechanisms by which oats or oat fractions lower cholesterol is not totally clear. Recognizing the important role of the gut microbiome in metabolism and metabolic disease risk, we examined the impact of whole grain oat Granola (WGO) on the human gut microbiota and cardio-metabolic risk factors using a randomized crossover dietary intervention in at risk individuals (ClinicalTrials.gov Identifier: NCT01925365). We randomized 32 individuals at risk of developing cardio-metabolic disease by virtue of mild hypercholesterolaemia or glucose intolerance, into two groups consuming either 45 g of WGO or non-whole grain (NWG) breakfast cereals daily for two 6-week intervention periods separated by a 4-week wash out period in a randomized, controlled, crossover, double-blinded design. Confirming the cholesterol lowering effect of WGO, we observed a significant time by treatment interaction, for total cholesterol (TC) (*P* = 0.0001) and LDL-cholesterol (LDL-C) (*P* = 0.02) compared to NWG. A significant time by treatment interaction was also observed for the relative abundance of fecal bifidobacteria (*P* = 0.0001), lactobacilli (*P* = 0.001) and total bacterial count (*P* = 0.008), which were all elevated after consumption of WGO. Daily consumption of WGO resulted in a prebiotic effect on the human gut microbiota composition and significant reductions in TC and LDL-C concentrations. Prebiotic modulation of the human gut microbiota may thus constitute a previously unrecognized mechanism contributing to the hypocholesterolaemic effects of whole grain oat Granola.

## Introduction

Several large epidemiological studies and a number of meta-analyses of nutritional interventions have reported a positive association between increased whole grain intake and reduced risk of developing a range of chronic diseases (Chatenoud et al., 1998; Jacobs et al., 1999; Montonen et al., 2003; Mellen et al., 2008; He et al., 2010; Ye et al., 2012). Consumption of oats or oat based products by individuals with various metabolic disease risk factors (e.g., hypercholesterolemia, obesity, and diabetes) and in different ethnic groups, has been shown to mediate an appreciable normalization of plasma cholesterol levels (Ripsin et al., 1992; Queenan et al., 2007; Wolever et al., 2011; Charlton et al., 2012; Zhang et al., 2012). The cholesterol lowering activity of oats is usually attributed to its ability to reduce intestinal absorption of cholesterol and/or inhibit the enterohepatic circulation of bile acids by increasing carriage of cholesterol and/or bile acids into the colon and facilitating their excretion in feces (Ryan et al., 2007; Gunness and Gidley, 2010; Borneo and León, 2012). Whole grain oats contain a number of potentially bioactive components capable of modulating cholesterol metabolism in mammals, including unsaturated fatty acids, fibers, such as beta-glucan, arabinoxylans, arabinogalactans, and resistant starch. Some of these polysaccharides can form viscous gels in aqueous solutions, and/or directly bind cholesterol or bile acids, while all are fermentable by the gut microbiota into short chain fatty acids (SCFA). Oats, and whole grain oat Granola also contains polyphenolic compounds and phytoeostrogens which may also modulate the gut microbiota and impact on host metabolic parameters (Ryan et al., 2007; Borneo and León, 2012). However, it is the gel forming nature of beta-glucans which is most commonly attributed to the cholesterol lowering effect of oats. Tiwari and Cummins (2011) performed a meta-analysis on 126 studies and concluded that there was a significant dose-dependent inverse relationship between beta-glucan from oats and barley and blood total cholesterol, LDL-cholesterol, and triacylglycerol concentrations with 3 g/day being sufficient to lower blood total cholesterol by −0.30 mmol/L (Tiwari and Cummins, 2011). While the ability of oats and beta-glucans to increase excretion of bile acids and cholesterol in feces appears to be well established, the underlying mechanism still remains to be fully elucidated. Although the gel forming nature of beta-glucans reducing bile acid/cholesterol absorption is the most commonly proposed mechanism, recent studies with probiotic microorganisms raise the possibility of bile salt hydrolase (BSH) activity as another possible mechanism by which oats and fermentable fibers can lower plasma cholesterol (Gunness and Gidley, 2010; Ooi et al., 2010; Ejtahed et al., 2011; Jones et al., 2012a,b). Studies on non-gel forming prebiotic fibers, which modulate gut bacteria and increase BSH active lactobacilli and bifidobacteria, support the hypothesis that an increased BSH activity due to gut bacterial modulation could reduce plasma cholesterol (Tanaka et al., 1999; Kim et al., 2004). Yet few studies have examined the impact of whole grain oat based food products on human gut microbiota and no study to date has measured the ability of dietary oats to modulate the composition and relative abundance of commensal bacteria *in vivo*.

The present study aimed to address this knowledge gap by determining the effectiveness of a whole grain oat (WGO) Granola breakfast cereal, compared to a refined breakfast cereal to beneficially modulate gut microbiota and its metabolic output, plasma lipids, gut satiety hormones and inflammation markers in a randomized, controlled, double-blind, crossover dietary intervention study. It tested the hypothesis that whole grain oat based cereal can mediate a prebiotic modulation of the human gut microbiota, particularly increased bifidobacteria abundance and concomitant reduction of plasma LDL cholesterol.

## Materials and methods

### Subjects

Men and women (age range 23–64 year), recruited from the general population, attended the Hugh Sinclair Unit of Human Nutrition, at the University of Reading in a fasted state for measurement of height, weight, plasma glucose, total cholesterol concentrations, and hematology. To qualify for entry into the study subjects required a BMI of 18–30 kg/m^2^ and either glucose intolerance (fasting glucose > 5.5 but < 6.9 mmol/L) or mild to moderate hypercholesterolaemic (total cholesterol > 5.2 but < 7.8 mmol/L). Exclusion criteria for the study were as follows: medical history of heart disease, diabetes mellitus, cancer, pancreatitis or renal disease, use of lipid lowering drugs, systemic corticosteroids or drugs for regulating hemostasis, exposure to any investigational agent < 42 d before the study; presence of gastrointestinal disorder or use of a drug likely to alter gastrointestinal motility or nutrient absorption, a history of substance misuse or alcoholism, a current pregnancy, planned pregnancy, or given birth in the past 12 months, antibiotic treatment 6 weeks previous to study start date, an allergy or intolerance to intervention breakfast cereals components or smoking. The study was conducted according to the guidelines laid down in the Declaration of Helsinki and was given a favorable ethical opinion for conduct by the University of Reading's Research Ethics Committee. All participants gave written informed consent before participation. The study was registered as a clinical trial on ClinicalTrial.gov (NCT01925365).

### Study design

The dietary intervention study was a randomized, double blind, controlled, crossover design. Thirty-two volunteers (20 women, 12 men) were recruited onto the study. For a 2-week period prior to dietary intervention, volunteers followed their habitual diet but were required not to consume confirmed prebiotics (such as inulin), probiotics (such as live yogurts or fermented milk drinks), and whole grain products. The subjects were randomly allocated into one of two groups using a random number generator. One group consumed the WGO Granola breakfast cereal (45 g/day) for 6 weeks, and then after a 4-week washout period, consumed the NWG breakfast cereal (45 g/day) for 6 weeks. The other group received the breakfast cereals in the opposite order. During each washout period no breakfast cereal was consumed. All test materials were packaged, labeled and randomized by Jordans Cereals (Biggleswade., UK) prior to the study, neither the investigators nor study subjects were aware of which cereal was allocated.

Study subjects were asked to keep diaries while ingesting the breakfast cereals, to record stool frequency and consistency (constipation, hard, formed, soft, or diarrhea), abdominal pain, intestinal bloating and flatulence (none, mild, moderate, or severe) on a daily basis. Any concomitant medication and adverse events were recorded. Faecal samples, saliva samples, and 20 ml fasting venous blood samples were collected from each volunteer at six time points before and after each treatment and 14 days after each of the treatment periods (i.e., 0, 42, 56, 70, 112, and 126 days).

### Composition of breakfast cereals

The nutritional composition of WGO and NWG breakfast cereals were provided by Jordans Cereals (Biggleswade., UK) except for the β-glucan content which was analyzed by Leatherhead Food Research (using the Megazyme kit). The energy content of the WGO and NWG cereals was 417 kcal/100 g and 380 kcal/100 g, respectively. WGO, in the form of Granola breakfast cereal, comprised mainly of whole grain oats with small amounts of almond and dried fruit, and contained (per 100 g) 67.8 g carbohydrates, of which 26.8 g sugars; 8.3 g protein, 12.4 g fat, 6.3 g fiber and 2.9 g β-glucan. NWG, in the form of flaked corn cereal, contained (per 100 g) 84.4 g carbohydrate, of which 8.8 g sugars; 7.4 g protein, 1.1 g fat, 3.0 g fiber and no detectable β-glucan.

### Culture independent enumeration of fecal bacteria

Fecal bacteria enumeration was the primary outcome measure. Fecal samples were stored in an anaerobic cabinet (10% H_2_; 10% CO_2_; 80% N_2_) for no longer than 2 h prior to processing. Changes in fecal bacteria populations upon consumption of the test and control cereals were monitored using 16S rRNA probes labeled with Cy3 for specific bacterial groups or the nucleic acid stain DAPI for total bacterial counts and Fluorescence *in situ* Hybridisation (FISH). The bacterial groups selected for enumeration were *Bifidobacterium* spp., *Bacteroides/Prevotella* spp., *Lactobacillus/ Enterococcus* spp., *Clostridium coccoides-Eubacterium rectale* group, *Clostridium histolyticum* group, and *Atopobium* cluster including most *Coriobacteriaceae* species, using the specific probes Bif164, Bac303, Lab158, Erec482, His150, and Ato291, respectively (Langendijk et al., 1995; Manz et al., 1996; Franks et al., 1998; Harmsen et al., 2000). FISH was performed essentially as described by Rycroft et al. (2001) and Daims et al. (2005). Briefly, fecal samples (375 μl) fixed in 4% paraformaldehyde (pH 7.2) overnight at 4°C were then centrifuged at 1500 × g for 5 min, washed twice with phosphate buffer saline (PBS 0.1 M, pH 7.0), re-suspended in a mixture of PBS/99% ethanol (1:1 v/v) and stored at −20°C for up to 3 months.

For the hybridisations, 20 μl of each sample was pipetted onto Teflon- and poly-l-lysine-coated, six-well (10 mm diameter each) slides (Tekdon Inc., Myakka City, FL, USA). Slides were dried at 46°C for 15 min and then submerged in a series of ethanol solutions (50, 80, and 96%, 3 min each). This process was used for all samples, except those where the Lab158 probe was used. Sample slides probed with Lab158 were subjected to an additional step with 50 μl of lysozyme (1 mg/mL in 100 mM Tris-HCl, pH 8.0) at 37°C for 15 min prior to being submerged in the ethanol solutions. A probe/hybridization buffer mixture (5 μl of a 50 ng μl^−1^ stock of probe plus 45 μl of hybridization buffer) was applied to the surface of each well. Hybridisations were performed for 4 h in an ISO20 oven (Grant Boekel). Slides were stored in the dark at 4°C (for a maximum of 3 days) until cells were counted. Slides were enumerated using a Nikon E400 Eclipse microscope fitted with an EPI-fluorescence attachment, 15 randomized views were counted for each sample.

### SCFA analysis

High-performance liquid chromatography (HPLC) was performed to determine fecal SCFA concentration. Aliquots (1 ml) of 10% (w/v) fecal suspension were centrifuged at 13000 g for 10 min and the supernatant was stored at −20°C for up to 3 months. Supernatants were then filtered using 0.2 mm polycarbonate syringe filters (Whatman, Maidstone, Kent, UK) and injected (20 μl) into an HPLC system (Merck, Whitehouse Station, NJ, USA) equipped with refractive index detection. For the preparation of the external standard containing the SCFA; acetic, propionic and butyric acid were added to give a final concentration of 25 mM to HCl (6 M) and HPLC gradient water. Dilutions of the external standards were prepared and added to the internal standard (ratio 4:1) to give a final concentration for the internal standard of 20 mM ethyl butyric acid, and a final concentration of external standards as 80, 40, 20, 10, 5.0, 1.0, and 0.5 mM. The column used was an ion-exclusion REZEX-ROA organic acid column (Phenomenex, Inc., Torrance, CA, USA) maintained at 85°C. H_2_SO_4_ in HPLC-grade H_2_O (0.0025 mmol/l) was used as the eluent, and the flow rate was maintained at 0.5 ml/min. Quantification of the samples was obtained through calibration curves of acetic, propionic and butyric acids.

### Blood samples collection and analysis

Blood samples were collected into a 10 ml EDTA tube (BD vacutainer EDTA tube, BD, Cowley, Oxon., UK) for the analysis of fasting total cholesterol (TC), HDL-cholesterol (HDL-C), LDL-cholesterol (LDL-C), triacylglycerol (TAG), CRP, IL-6, TNF-α, PYY, GLP-1 and insulin concentrations; into a 10 ml fluoride/oxalate tube (BD vacutainer fluoride/oxalate tube) for the analysis of fasting glucose concentration. These blood analytes were the secondary outcome measures. Following collection all blood samples were kept on ice until centrifugation. Plasma samples were recovered by centrifugation at 1700 g for 10 min, dispensed into 1.5 ml microcentrifuge tubes and frozen at −20°C within 1 h from collection. Plasma samples were defrosted and centrifuged for 5 min at 1500 g prior to analysis.

Plasma TAG, glucose, CRP, TC, LDL-C, and HDL-C concentrations were determined on a Monarch Automatic Analyzer ILab 600 using enzymatic kits (Instrumentation Laboratories Ltd, Cheshire, UK). Two quality control samples, (Wako Control Serum I and II. Alpha Laboratories Ltd, Eastleigh, Hants., UK), containing known normal and abnormal concentrations were included at the beginning and end of each batch analysis. Results were accepted if the quality control values were within the range specified by the manufacturers.

Determination of plasma insulin (DAKO Diagnostic Ltd, Cambridgeshire, UK), IL-6 and TNF-α (R&D Systems, Abingdon, UK), PYY and GLP-1 (Yanaihara Institute Inc. Shizuoka, Japan), feacal and saliva sIgA (Immundianostik AG, Bensheim, Germany) calprotectin (NovaTec Immundiagnostica GmbH, Dietzenbach, Germany) concentrations were performed using specific commercial ELISA kits according to manufacturer's instructions. Concentrations of the samples and quality controls were determined automatically by reading from the standard curve using MasterPlex (version EX 2010) computer software.

### Dietary analysis

Volunteers were asked on week 5 of each of the intervention arms to complete a 3-day estimated diet diary (2 week and one weekend day). The volunteers received both verbal and written instructions on how to complete the diet diaries. These were analyzed using Dietplan 6.60 (Forestfield Software Ltd), to determine the macro and micronutrient content of the participant's diets during each intervention period.

### Statistical analysis

Normality testing was carried out to determine if data was normally distributed and transformed if required. Linear mixed model analysis of variance was performed for testing effects of visit, treatment, and visit by treatment interaction. Tukey's post-test with 95% confidence was used for multiple comparisons after a visit by treatment interaction was established. Minitab 16 for Windows was used for all analysis. *P* <0.05 was taken as significant. The sample size of 28 was required to detect 0.5 Log_10_ change in bifidobacterial counts with power set at 0.9, and a significance level of 0.05 based on previous prebiotic studies in healthy volunteers conducted with the same microbiological techniques investigating whole grain maize (Carvalho-Wells et al., 2010). To allow for a 10% dropout rate a total of 32 individuals were recruited.

## Results

### Subjects characteristics

Of the 32 participants recruited, one individual withdrew before the start of the study due to antibiotic treatment, and another withdrew after visit 1 due to personal reasons. Samples from these individuals were excluded from analysis. Thus, a total of 30 participants were included (women *n* = 19, men *n* = 11). The mean age was 42 years with a range between 19 to 60 years. The participant's baseline mean weight was 75.2 kg (SD ± 20.4), BMI 26.4 (± 5.7), were mildly hypercholesterolamic TC 5.4 mmol/L (± 1.0) or glucose intolerant (glucose 5.6± 0.6 mmol/L).

### Dietary analysis

Nutrient analysis of the diets during the two intervention periods is shown in Table 1. The nutrient intakes in both intervention periods were similar except for total fat as % of total food energy (%E) (*P* = 0.009) and MUFA (%E) (*P* = 0.002) which were higher, and starch (%E) (*P* = 0.007), which was lower while participants consumed the WGO compared with the NWG cereal. These differences broadly reflected compliance to the intervention foods.

**Table 1:** **Mean (SEM) of daily macronutrient intake during the WGO and NWG intervention periods^**a**^**.

**Daily intake (*n* = 24)**	**WGO treatment Mean ± SEM**	**NWG treatment mean ± SEM**	***P*-value**
Energy, *kJ*	7904±404	7512±349	0.312
Energy, *kcal*	1916±78	1787±83	0.301
Fat, *%E*	34.0±1.1	30.6±1.0	0.009
SFA, *%E*	11.9±0.5	10.9±0.7	0.162
MUFA, *%E*	12.5±0.6	10.4±0.5	0.002
PUFA, *%E*	5.9±0.4	5.5±0.4	0.238
Total TFA, *%E*	0.7±0.1	0.8±0.1	0.158
Protein, *%E*	16.4±0.8	17.0±0.8	0.407
Carbohydrate %E	49.8±1.3	52.3±1.2	0.073
Starch, *%E*	27.1±1.2	31.4±1.4	0.007
Total Sugar, *%E*	21.2±1.1	18.7±1.4	0.080
NSP, *g*	14.4±0.7	13.9±0.9	0.615
Total fiber (AOAC method), *g*	18.8±0.9	18.2±1.1	0.626

a *Determined from estimated 3-day dietary diaries. SFA, saturated fatty acids; MUFA, monounsaturated fatty acids; PUFA, polyunsaturated fatty acids; TFA, trans fatty acids; NMES, non-milk extrinsic sugars; NSP, non-starch polysaccharide; AOAC, Association of Official Analytical Chemists*.

### Faecal bacteria and SCFA analysis

**Figure 1:**
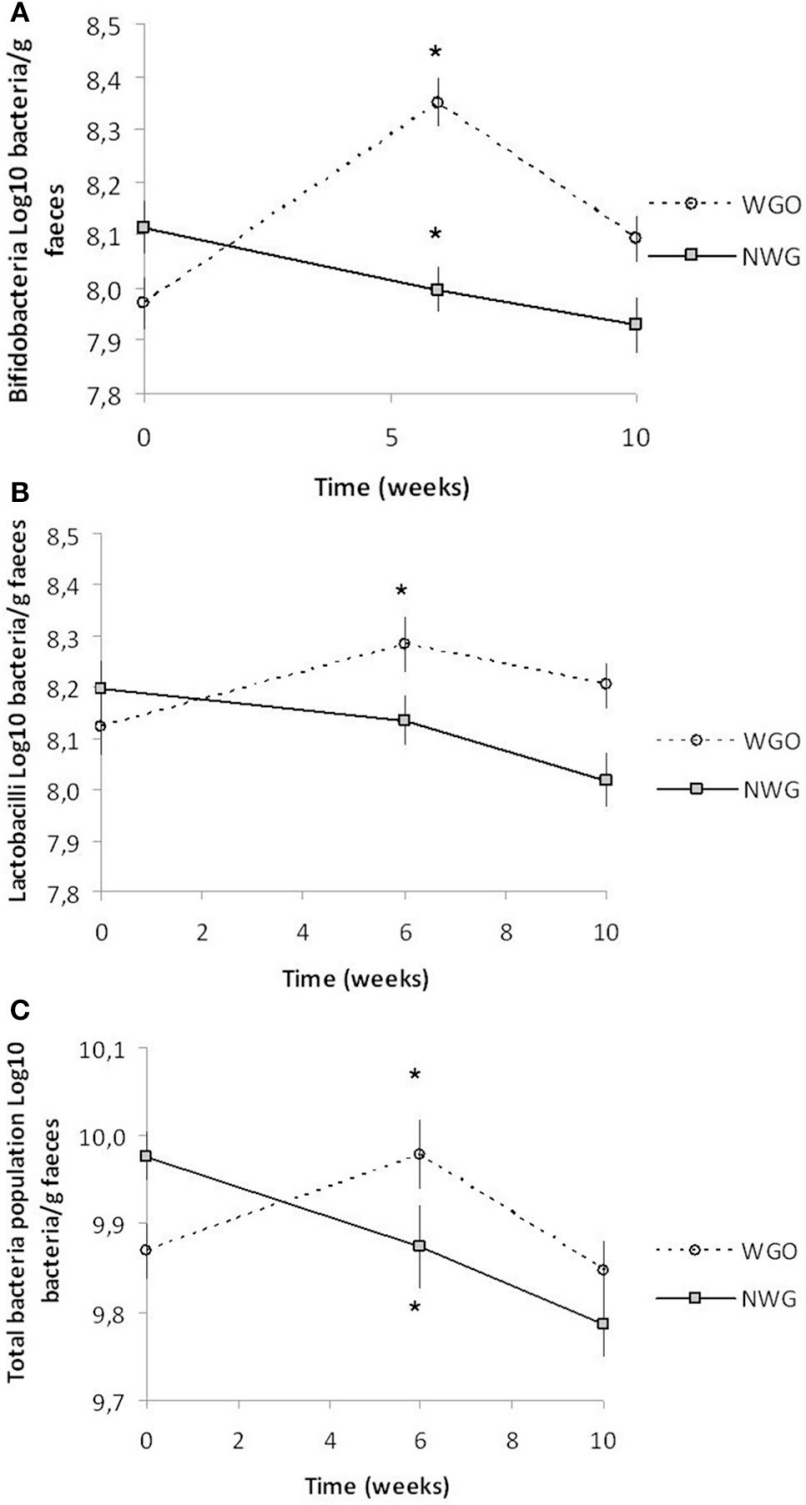
**Mean (± SEM) fecal bacteria changes over the trial period for both cereal treatments (***n*** = 30). (A)** Bifidobacteria, **(B)** Lactobacilli, and **(C)** Total Population in stool samples as determined by fluorescence *in situ* hybridization. ^*^ Significantly different from baseline (*P* <0.05, Tukey's post-test).

The data for the fecal SCFA analysis is shown in Table 2. No significant changes were detected either between visits or groups. Fluorescence *in situ* hybridization was used to enumerate the main gut bacterial groups (Table 3). Baseline levels of all bacterial groups were comparable between the two treatments. A time by treatment interaction for bifidobacteria (*P* = 0.000; Figure 1A), lactobacilli (*P* = 0.001; Figure 1B) and total bacterial population (*P* = 0.008; Figure 1C) was observed. After consumption of WGO for a 6-week period the numbers of bifidobacteria (Log_10_ bacteria cells/g feces) (*P* = 0.000), lactobacilli (*P* = 0.000), and total bacteria population (*P* = 0.017) significantly increased compared to the respective baseline. A significant decrease in bifidobacteria (*P* = 0.03) and total bacteria population (*P* = 0.034) was observed on NWG consumption after the 6-week feeding time. A significant difference at 6 weeks between bifidobacteria (*P* = 0.000) and total population (*P* = 0.003) size after WGO and NWG was observed, however they returned to near baseline levels after the 4-week washout. No significant changes in the population size of *Bacteroides* spp., *Atopobium* spp., and *Clostridium* group were observed.

**Table 2:** **Mean (±SEM) concentrations of fecal short chain fatty acids (SCFA) before during and after WGO and NWG treatment arms**.

	**Pre-WGO**	**Post-WGO**	**Wash-WGO**	**Pre-NWG**	**Post-NWG**	**Wash-NWG**	***P*-value**
**SCFA**	**Mean ± SEM**	**Mean ± SEM**	**Mean ± SEM**	**Mean ± SEM**	**Mean ± SEM**	**Mean ± SEM**	**Time by treat**
Acetate, mM/g	53.7±12.5	67.4±12.9	70.3±15.2	59.4±15.9	66.8±19.1	57.2±18.4	0.213
Propionate, mM/g	6.5±2.5	21.5±4.1	19.5±3.2	21.3±3.5	24.5±4.9	24.3±5.5	0.572
Butyrate, mM/g	14.6±2.0	18.7±5.2	22.1±4.5	13.4±3.1	18.2±5.3	15.7±4.6	1.33

*(n = 30), visit 1 represents baseline*.

**Table 3:** **Mean (± SEM) fecal bacterial numbers (***n*** = 30) over the trial period**.

	**Pre-WGO**	**Post-WGO**	**Wash-WGO**	**Pre-NWG**	**Post-NWG**	**Wash-NWG**	***P*-value**
**Bacteria Group, log_10_ cells/g feces**	**Mean ± SEM**	**Mean ± SEM**	**Mean ± SEM**	**Mean ± SEM**	**Mean ± SEM**	**Mean ± SEM**	**Time by treat**
*Bifidobacterium*	7.97 ± 0.05^a^	8.35 ± 0.05^a^^b^	8.09 ± 0.05^a^	8.12 ± 0.05^b^^c^	8.00 ± 0.04^b^	7.93 ± 0.05^b^^c^	0.000
*Bacteroides* and *Prevotella*	8.97 ± 0.05	8.96 ± 0.05	8.95 ± 0.05	8.96 ± 0.05	8.97 ± 0.05	8.94 ± 0.05	0.990
*Lactobacillus*	8.12 ± 0.06^a^^b^	8.28 ± 0.06^a^^b^^e^	8.20 ± 0.05^a^^b^^f^	8.20 ± 0.05^c^	8.14 ± 0.05^d^	8.02 ± 0.05^c^^d^^e^^f^	0.001
*Ruminococcus*	8.77 ± 0.06	8.89 ± 0.06	8.83 ± 0.07	8.85 ± 0.06	8.70 ± 0.07	8.67 ± 0.07	0.059
*Clostridium histolyticum/perfringens*	7.98 ± 0.07	7.92 ± 0.06	7.92 ± 0.06	7.95 ± 0.05	7.98 ± 0.06	7.95 ± 0.07	0.632
*Atopobium*	8.01 ± 0.06	7.97 ± 0.06	8.00 ± 0.05	8.01 ± 0.06	8.05 ± 0.06	8.09 ± 0.05	0.618
*Total Population*	9.87 ± 0.03^a^	9.98 ± 0.04^a^^b^^e^	9.85 ± 0.03^b^	9.98 ± 0.03^c^^d^	9.87 ± 0.05^c^	9.78 ± 0.04^d^^e^	0.008

Bacterial counts in stool samples as determined by fluorescence in situ hybridisation are shown expressed as mean log_10_ cells/g feces. WGO, Whole Oat Grain; NWG, Non-Whole Grain;

**abcdef:** *All values in one row with a common letter are significantly different from each other (P < 0.05, Tukey's post-test)*.

### Blood lipid parameters

**Figure 2:**
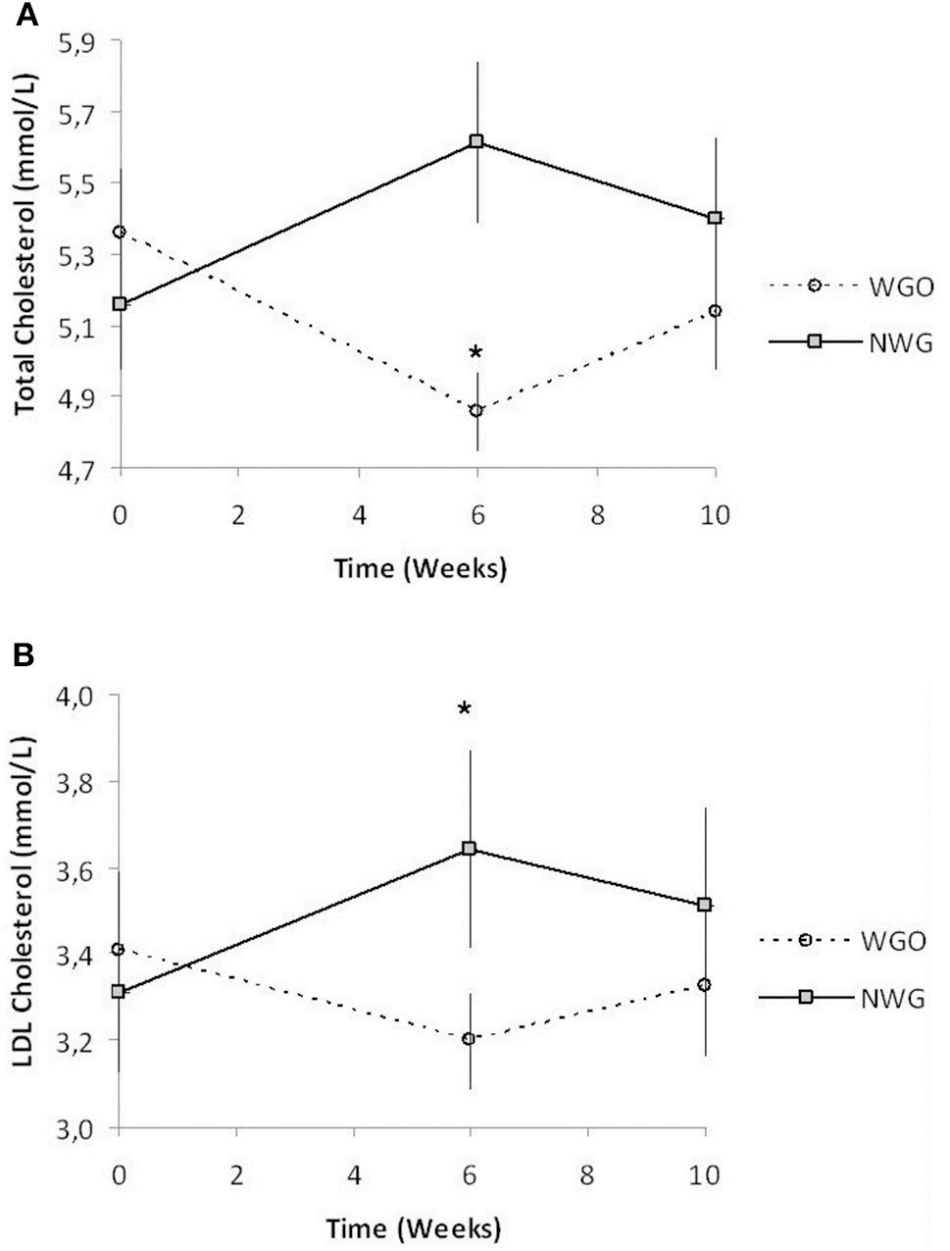
**TC and LDL-C concentrations in fasted blood plasma over the trial period for both cereal treatments (***n*** = 30). (A)** TC and **(B)** LDL-C. ^*^ Significantly different from baseline (*P* <0.05, Tukey's post-test).

Baseline concentrations of all lipid parameters were comparable between the two treatments (Table 4). A significant time by treatment interaction for TC (*P* = 0.000), and LDL-C (*P* = 0.002) was observed. After consumption of WGO for a 6-week period, TC concentrations significantly decreased (*P* = 0.0016), in contrast after NWG a significant increase in TC levels was observed (*P* = 0.0016; Figure 2A). Plasma LDL-C was seen to significantly increase (*P* = 0.0055) after consumption of the NWG cereal (Figure 2B). A significant difference at 6 weeks between TC (*P* = 0.000) and LDL-C (*P* = 0.009) after WGO and NWG was observed, however they returned to near baseline levels after the 4-week washout. No significant changes in HDL-cholesterol, glucose or TAG concentrations were observed.

**Table 4:** **Mean (±SEM) fasting concentrations of plasma lipids and lipoprotein concentrations, glucose, insulin, markers of insulin resistance, and inflammatory markers at baseline, during and following ingestion of WGO and NWG**.

	**Pre-WGO**	**Post-WGO**	**Wash-WGO**	**Pre-NWG**	**Post-NWG**	**Wash-NWG**	***P*-value**
**Biomarker**	**Mean ± SEM**	**Mean ± SEM**	**Mean ± SEM**	**Mean ± SEM**	**Mean ± SEM**	**Mean ± SEM**	**Time by treat**
TC, *mmol/L*	5.38±0.18	4.86±0.11∞	4.99±0.17	5.31±0.19	5.80±0.23∞	5.4±0.24	0.000
HDL, *mmol/L*	1.46±0.08	1.48±0.08	1.52±0.08	1.51±0.08	1.50±0.08	1.43±0.07	0.158
LDL, *mmol/L*	3.41±0.92	3.22±1.14	3.18±1.08	3.31±0.16	3.62±0.14∞	3.51±0.15	0.002
TAG, *mmol/L*	1.21±0.11	1.13±0.11	1.06±0.11	1.38±0.20	1.46±0.20	1.38±0.20	0.518
Glucose, *mmol/L*	5.44±0.10	5.33±0.19	5.33±0.12	5.71±0.13	5.82±0.11	5.88±0.12	0.066
Insulin, *ulU/mL*	8.42±0.92	8.66±1.13	7.72±1.00	7.91±1.01	10.23±1.47	8.95±1.08	0.296
HOMA-IR ∇	2.17±0.27	2.21±0.32	2.00±0.31	1.99±0.27	2.63±0.39	2.31±0.30	0.259
QUICKI ∇	0.35±0.01	0.36±0.01	0.36±0.01	0.36±0.01	0.35±0.01	0.35±0.01	0.137
CRP, *mg/L*	1.69±0.35	2.45±0.92	1.82±0.47	1.80±0.47	2.36±0.49	2.02±0.48	0.934
IgA Saliva, *ng/mL*	265±27	245±44	288±45	229±24	340±47	299±41	0.208
IgA Stool, *ng/mL*	157±31	154±39	156±36	162±31	173±42	194±48	0.197
TNF-alpha, *pg/mL*	20.2±4.0	36.5±15.7	32.9±10.9	46.3±26.0	42.2±14.8	27.4±8.4	0.519
Calprotectin, *mg/kg*	25.2±8.2	25.9±9.6	20.0±4.9	24.5±8.9	27.6±10.3	24.9±8.4	0.066
IL-6, *pg/mL*	4.13±1.47	5.88±178	3.85±2.00	4.09±1.71	7.16±3.46	4.85±1.74	0.925
PYY, *ng/mL*	0.88±0.07	0.79±0.08	0.93±0.07	0.77±0.06	0.78±0.03	0.91±0.06	0.551
GLP-1, *ng/mL*	1.04±0.04	1.01±0.06	1.12±0.03	1.01±0.04	1.02±0.03	1.08±0.03	0.641
BMI, *kg/m^2^*	26.3±1.1	26.1±1.1	26.2±1.1	26.2±1.0	26.3±1.0	26.3±1.0	0.061
Fat Mass, *kg*	23.0±2.0	22.6±2.0	22.8±2.0	22.8±1.9	23.2±2.0	22.9±1.2	0.059

*BMI, fat mass and weight and concentrations of satiety gut hormones GLP-1 and PYY of participants before, after and following washout of both test cereals (n = 30)*.

*^∞^Significantly different from baseline (P < 0.05, Tukey's post-test)*.

*∇ Insulin sensitivity calculated using Quantitative Insulin Sensitivity Check Index (QUICKI), and insulin resistance calculated using homeostatic model assessment (HOMA) measured before and after 42 wk of either WGO or NWG treatment. All values are average of volunteer's measurements ± SEM, (n = 30). All measurements were taken in the morning after the subjects had fasted for 12 h*.

### Insulin sensitivity/resistance and marker of inflammation

The homeostasis model for insulin resistance (HOMA IR), as well as QUICKI (model for insulin sensitivity) was calculated for both treatments (Table 4). There was no significant effect for either treatment. However, there was a near significant time by treatment interaction for fasting glucose (*p* = 0.066). After the WGO cereal fasting plasma glucose reduced and was lower at the end of treatment (week 6) than after the NWG. Biomarkers of inflammation including; C-Reactive Protein (CRP), IL-6, TNF-alpha from blood plasma, IgA in stool and saliva and calprotectin levels in stool samples are shown in Table 4. No significant time by treatment interaction was recorded for any of the inflammation biomarkers after consumption of either cereal treatments.

### Anthropometric measures and satiety gut hormones

The data for changes in volunteer's GLP-1 and PYY concentrations and BMI, weight and fat mass are shown in Table 4. No significant changes were detected between visits or groups for either of the cereal treatments.

### Biomarkers of gut health

Stool frequency and consistency, qualitatively graded by volunteers as hard, formed, or soft, varied considerably between individuals. No significance differences were observed between treatments. The severity and frequency of reported changes in digestive tolerance varied greatly between volunteers, with neither treatment resulting in adverse symptomology (data not shown).

## Discussion

This novel study was designed to test the hypothesis that a whole grain oat based Granola breakfast cereal can mediate a prebiotic modulation of the human gut microbiota typified by increased relative abundance of bifidobacteria in particular, and concomitant reduction of plasma LDL cholesterol. These data also support a significant body of evidence that ingestion of oats, and oat derived fractions, most notably, β-glucan, at 3 g/day, is associated with a significant reduction in blood TC and LDL-C in hypercholesterolemic groups (EFSA Panel on Dietetic Products, Nutrition and Allergies (NDA), 2010; Tiwari and Cummins, 2011). Although, the bile acid and possibly cholesterol binding abilities of β-glucans have been suggested to be responsible for the hypocholesterolemic effects *in vivo*, other mechanisms may also be involved, including those linked to the human gut microbiota which have not been addressed to date. In this study the WGO group consumed 1.3 g β-glucan daily, which is under half of the daily recommendation for significant TC and LDL-C reduction. However, despite the low β-glucan dose, WGO consumption resulted in 0.94 mmol/l and 0.4 mmol/l lower concentrations of TC and LDL-c, respectively, compared with the NWG at the end of the 6-week intervention period. These data suggest that other mechanisms of action, other than β-glucan, could have contributed to the observed cholesterol reduction. Similar to β-glucan, other fibers or prebiotics, which do not form viscous gels within the intestinal tract, have been shown to lower blood cholesterol, though these prebiotics including the fructans, xylooligosaccharides, galactooligosaccharides and resistant starch have not received the same attention from the scientific community as β-glucans (Park et al., 2004; Beylot, 2005; Sheu et al., 2008; Vulevic et al., 2013). In parallel, a number of human feeding studies with well-powered cohorts of hypercholesteroleamic individuals have demonstrated that probiotics selected for bile salt hydrolase (BSH) activity lower plasma cholesterol levels to a similar extent to oats and β-glucan (Jones et al., 2012a,b). Such observations raise the intriguing possibility, that along with the viscous gel forming activities of β-glucan, oats may also possess other biological activities which could contribute to their cholesterol lowering ability; not least the ability to stimulate bacteria within the gut with BSH activity thereby increasing bile acid deconjugation, facilitating bile acid excretion in feces and triggering *de novo* hepatic bile acid synthesis from circulating cholesterol. Therefore, the current study was designed to investigate whether WGO mediate a prebiotic modulation of the human gut microbiota with specific increases in bifidobacteria, a group of bacteria with well recognized BSH activity and whether this was associated with reducing plasma cholesterol (Tanaka et al., 1999).

Currently few studies, and none containing oats, have measured the ability of whole grain cereals to modulate bacterial relative abundance in human feces (Costabile et al., 2008; Carvalho-Wells et al., 2010). Andersson et al. (2013) observed gut microbiota modulation in response to a dietary hypocholesterolic intervention with oats in two strains of C57BL/6 mice. We have previously shown that different whole grain cereals, including whole grain oat preparations, have prebiotic potential *in vitro*, determined by significant stimulation of both bifidobacteria and lactobacilli bacteria (Connolly et al., 2010, 2012). Using the same WGO cereal that induced the highest modulation in bifidobacteria and lactobacilli bacteria *in vitro* and also had the lowest GI (37), we have shown for the first time in humans that WGO consumption for 6 weeks can stimulate a prebiotic modulation of the gut microbiota selectively increasing fecal bifidobacteria, lactobacilli, and total bacteria compared to a control treatment (Connolly et al., 2010, 2012). These bacterial numbers returned to baseline levels after 4 weeks washout in the group of metabolically at risk participants. No significant changes in *Bacteroides* spp., *C. histolyticum/perfringens* group, or *Atopobium* spp. was detected in the fecal samples collected, as also found in previous studies with other prebiotics. Furthermore, we observed a significant reduction in TC and LDL-C concentrations after the WGO treatment compared with NWG supporting other findings (EFSA Panel on Dietetic Products, Nutrition and Allergies (NDA), 2010; Tiwari and Cummins, 2011). Although this was associated with a significantly higher monounsaturated fatty acid (MUFA) and lower starch intake in the WGO group, this small absolute change in macronutrient intake is unlikely to have impacted on the circulating lipids, based on the predicted changes reported by Mensink et al. (1% dietary energy exchange of carbohydrate with MUFA was associated with a non-significant reduction in total cholesterol of 0.006 mmol/l and a significant, but relatively small reduction in LDL-C of 0.009 mmol/l) (Mensink et al., 2003). A number of well controlled animal and human feeding studies with strains of bacteria which possess bile salt hydrolase (BSH) activity, like *Bifidobacterium* and *Lactobacillus* spp., have reported significant reductions in circulating TC and LDL-C levels to a similar degree as oats, β-glucans and other gel forming fibers like psylleum (Begley et al., 2006; Ooi et al., 2010; Ejtahed et al., 2011; Jones et al., 2012a,b). In addition *in vitro* fermentation studies have shown that various oat fractions, including β-glucans can both increase SCFA production and modulate relative abundance of key gut microbiota bacteria (Hughes et al., 2008; Kim and White, 2009; Connolly et al., 2012; Nordlund et al., 2012). The ability of oats and β-glucans to increase SCFA production by the gut microbiota *in vitro* and in animal studies has been shown, while few studies have examined the ability of oats or β-glucans to increase fermentation in humans (Nilsson et al., 2008; Turunen et al., 2011). It has been postulated that possible mechanisms of action of prebiotics on cholesterol reduction may include physiological actions of SCFA, particularly propionate, to reduce *de novo* hepatic cholesterol synthesis by down regulation of HMG CoA reductase; increased cholesterol excretion from cholesterol assimilation, deconjugation of bile acids and cholesterol binding to bacterial or plant cell walls (Shapiro and Rodwell, 1971; Levrat et al., 1994).

The mechanisms involved with the observed cholesterol reduction in the present study are not clear. A possible limitation of the study was that plasma bile salts and SCFA were not measured. Although it was observed that fecal SCFA concentrations were similar between treatments, these data may not reflect plasma SCFA concentrations and this mechanism cannot be ruled out. Recent studies have shown that SCFA induce their own active uptake transporters on the gut wall, hindering the use of fecal SCFA concentrations as an indicator of colonic SCFA production from fermentation (Borthakur et al., 2012). Further mechanistic studies are required to examine how prebiotic modulation of the gut microbiota may be linked to cholesterol metabolism, and specifically whether a prebiotic type microbiota modulation increases bile acid deconjugation impacting on bile acid excretion or signaling within the intestine and at other body sites specifically in the liver and brain. In summary we have confirmed that dietary WGO ingestion had an appreciable impact on the composition of the human gut microbiota, and significantly reduces plasma TC and LDL-C, a step toward understanding their gut mediated communication with host energy metabolism.

## Author contributions

JL, KT, and MC designed the study. MC conducted the study. MC analyzed the data and drafted the paper and XT performed the dietary analysis. JL, KT, and XT edited the paper and all authors have read and approved the final manuscript. JL and KT had primary responsibility for final content.

## Funding

The study was funded by the Reading Endowment Trust Fund (RETF) and Jordans Cereals (Biggleswade, UK) who kindly supplied the breakfast cereals, however the sponsor had no input into the study hypothesis and design, data analysis and interpretation.

### Conflict of interest statement

The authors declare that the research was conducted in the absence of any commercial or financial relationships that could be construed as a potential conflict of interest.
